# Whole-Genome Sequencing to Determine Origin of Multinational Outbreak of *Sarocladium kiliense* Bloodstream Infections

**DOI:** 10.3201/eid2203.151193

**Published:** 2016-03

**Authors:** Kizee A. Etienne, Chandler C. Roe, Rachel M. Smith, Snigdha Vallabhaneni, Carolina Duarte, Patricia Escandón, Elizabeth Castañeda, Beatriz L. Gómez, Catalina de Bedout, Luisa F. López, Valentina Salas, Luz Maria Hederra, Jorge Fernández, Paola Pidal, Juan Carlos Hormazabel, Fernando Otaíza-O’Ryan, Fredrik O. Vannberg, John Gillece, Darrin Lemmer, Elizabeth M. Driebe, David M. Engelthaler, Anastasia P. Litvintseva

**Affiliations:** Centers for Disease Control and Prevention, Atlanta, Georgia, USA (K.A. Etienne, R.M. Smith, S. Vallabhaneni, A.P. Litvintseva);; Georgia Institute of Technology, Atlanta (K.A. Etienne, F.O. Vannberg);; Translational Genomics Research Institute, Flagstaff, Arizona, USA (C.C. Roe, J. Gillece, D. Lemmer, E.M. Driebe, D.M. Engelthaler);; Instituto Nacional de Salud, Bogota, Colombia (C. Duarte, P. Escandón, E. Castañeda);; Universidad del Rosario, Bogota (B.L. Gómez);; Corporación para Investigaciones Biológicas, Medellín, Colombia (B.L. Gómez, C. de Bedout, L.F. López);; Instituto de Salud Publica de Chile, Santiago, Chile (V. Salas, L.M. Hederra, J. Fernández, P. Pidal, J.C. Hormazabel);; Ministry of Health, Santiago (F. Otaíza-O’Ryan)

**Keywords:** fungal outbreaks, epidemiology, whole-genome sequencing, fungi, Latin America, bloodstream infections, Chile, Colombia

## Abstract

We used whole-genome sequence typing (WGST) to investigate an outbreak of *Sarocladium kiliense* bloodstream infections (BSI) associated with receipt of contaminated antinausea medication among oncology patients in Colombia and Chile during 2013–2014. Twenty-five outbreak isolates (18 from patients and 7 from medication vials) and 11 control isolates unrelated to this outbreak were subjected to WGST to elucidate a source of infection. All outbreak isolates were nearly indistinguishable (<5 single-nucleotide polymorphisms), and >21,000 single-nucleotide polymorphisms were identified from unrelated control isolates, suggesting a point source for this outbreak. *S. kiliense* has been previously implicated in healthcare-related infections; however, the lack of available typing methods has precluded the ability to substantiate point sources. WGST for outbreak investigation caused by eukaryotic pathogens without reference genomes or existing genotyping methods enables accurate source identification to guide implementation of appropriate control and prevention measures.

## Abstract 2

Next-generation technologies and bioinformatics enabled source attribution and implementation of effective control strategies.

Despite modern advances in technology to control fungal contamination in clinical settings, fungi are continuously implicated in clusters or outbreaks of infections, particularly among immunosuppressed patients (*1*). The sources of fungal nosocomial outbreaks often are difficult to assess because of the widespread prevalence of fungi in the environment. Specifically, differentiating between fungal infections originating from a single contaminated point source and those independently acquired from the environment frequently is difficult. Molecular typing methods for discriminating strains have been an essential tool to identify potential source(s) of fungal infections in outbreaks. Small-scale DNA-based typing methods, such as variable-number tandem-repeat typing and multilocus sequence typing (MLST), use genomic similarity to assist in determining epidemiologic relatedness of fungal isolates in an outbreak investigation (*2*). However, the robustness and accuracy of such genotyping tools depend largely on the discriminatory power of the genotyping method and availability of reference data, which often are inconsistent or incomplete for many fungi. 

The advent of whole-genome sequence typing (WGST) has made fungal genotyping feasible for outbreak investigations (*3*–*7*), especially for fungi for which conventional genotyping methods do not exist. With WGST, genetic relationships among isolates are determined by the phylogenetic analysis of single-nucleotide polymorphism (SNP) differences among analyzed genomes a population: typically, the fewer the number of SNPs observed between the strains, the more closely the strains are related, and the more likely they are to have a point source, provided supporting epidemiologic evidence exists. Point source outbreaks are typically clonal, and the resulting isolate genomes display few to no SNP differences. Environment-linked outbreaks might have 1 or multiple source populations that also are identifiable and distinct from background and control strains (*5*).

In January 2014, the Chilean Ministry of Health contacted the Mycotic Diseases Branch (in the Division of Foodborne, Waterborne, and Environmental Disease, National Center for Emerging and Zoonotic Infectious Diseases), US Centers for Disease Control and Prevention (CDC), about a cluster of 67 cases of *Sarocladium kiliense* (formerly *Acremonium kiliense*) bloodstream infections (BSI). This cluster was identified at 8 different hospitals in Santiago, Chile, by the National Infection Control Program. The infections occurred during June 2013–January 2014; 39 infections were in children and 2 in adults, all of whom were undergoing chemotherapy. The Chilean Ministry of Health initiated an epidemiologic investigation with technical assistance from CDC and the Pan American Health Organization. An environmental source was considered unlikely because of the spread of these infections among multiple locales in Chile; however, the possibility of environmental contamination could not be excluded.

A detailed review of medication administration records revealed that all patients received 4 intravenous medications: ondansetron, heparin, saline, and potassium. Heparin, saline, and potassium were products used in many different patient populations within the hospital. However, ondansetron, an antinausea medication, was used as standard protocol among oncology patients, and all patients with *S. kiliense* BSIs received ondansetron from a single source, pharmaceutical company A, in Colombia. Three lots of ondansetron, manufactured by pharmaceutical company A, were investigated in Chile Drug National Agency (ANAMED, ISP). In accordance with Mycotic Diseases Branch recommendations (S. Vallabhaneni, pers. comm.) the Chilean Ministry of Health laboratory cultured 10% of unopened ondansetron vials of these 3 lots. Vials from 2 of the 3 available lots yielded *S. kiliense* on February 15, 2014. All isolates were identified by traditional methods and confirmed by DNA sequencing by ISP. After this finding, all ondansetron products made by this manufacturer were recalled in Chile; the Pan American Health Organization issued an international health alert on February 17, 2014.

Concurrently, the Corporación para Investigaciones Biológicas (Medellin, Colombia) and the Instituto Nacional de Salud (Bogota, Colombia) contacted CDC about an isolate identified as *S. kiliense* by conventional DNA sequencing methods and 16 isolates originally identified as a *Fusarium* spp. by phenotypic methods in Colombia dating to November 2013. Because of the findings in Chile, these isolates were reevaluated and confirmed as *S. kiliense* by conventional DNA sequencing methods (*8*). Further investigation by officials in Colombia showed that at least 14 of the 16 patients also received ondansetron manufactured by pharmaceutical company A in Colombia; culturing and conventional DNA sequence identification methods also confirmed that ondansetron was contaminated with *S. kiliense*.

A subset of isolates from patients and medication vials in this investigation were sent to CDC’s Mycotic Diseases Branch for further identification and molecular typing. To determine whether the contaminated lots of ondansetron harbored the same fungal strains as infected patients, we used WGST.

## Methods

### Isolates

On the basis of the availability of epidemiologic and patient information, we subjected a subset of patient isolates from each country to molecular analysis: 7 isolates from Chile and 11 isolates from Colombia, 1 isolate per patient. Additionally, 7 isolates from contaminated ondansetron vials were collected from both countries (Table). Eleven unrelated *S. kiliense* control isolates from the CDC culture collection, American Type Culture Collection, Centraalbureau voor Schimmelcultures, and Universitat Rovira I Virgili also were included for analysis. No background isolates of *S. kiliense* from the affected countries were available for analysis. Genomic DNA was extracted from cells grown on Sabouraud dextrose agar by using the DNeasy Blood and Tissue kit (QIAGEN, Hilden, Germany) as referenced in Litvintseva et al. (*3*).

**Table:** Whole-genome sequenced strains of *Sarocladium kiliense*, Chile and Colombia, 2013–2014*

Laboratory identification	Type of isolate	Source of isolate	Origin of isolate	Depth of coverage, ×
B10646	Patient	Chile	Blood	228
B10648	Patient	Chile	Blood	218
B10650	Patient	Chile	Blood	72
B10651	Patient	Chile	Blood	193
B10652	Patient	Chile	Blood	119
B10653	Patient	Chile	Blood	67
B10657	Patient	Chile	Blood	354
B10660	Ondansetron	Chile	Vial	78
B10661	Ondansetron	Chile	Vial	29
B10731	Patient	Colombia	Blood	75
B10732	Patient	Colombia	Blood	34
B10734	Patient	Colombia	Blood	67
B10743	Patient	Colombia	Blood	37
B10748	Ondansetron	Colombia	Vial	98
B10749	Ondansetron	Colombia	Vial	152
B10762	Patient	Germany	Skin lesion	54
B10763	Patient	Utah, USA	Eye	41
B10764	Patient	Wisconsin, USA	Skin lesion	43
B10765	Patient	Pennsylvania, USA	Blood	27
B10766	Patient	Florida, USA	CSF	74
B10767	Patient	Texas, USA	BAL	51
B10971	Patient	Colombia	Blood	101
B10972	Patient	Colombia	Blood	95
B10973	Patient	Colombia	Blood	62
B10974	Patient	Colombia	Blood	103
B10975	Patient	Colombia	Blood	113
B10976	Patient	Colombia	Blood	191
B10977	Patient	Colombia	Blood	261
B10978	Ondansetron	Colombia	Vial	150
B10979	Ondansetron	Colombia	Vial	126
B10980	Ondansetron	Colombia	Vial	152
B5504	Patient	Pennsylvania, USA	Eye	138
B5505	Patient	Pennsylvania, USA	Eye	38
ATCC64672	Dog	Costa Rica	Eye	26
CBS155	Environment	India	Soil	100
CBS157	Environment	India	Soil	82

*ATCC, American Type Culture Collection; CBS, Centraalbureau voor Schimmelcultures.

### Library Preparation and Illumina Sequencing

The 36 DNA samples were prepared for Illumina sequencing (Illumina, San Diego, CA, USA) by using the KAPA Biosystems Library Preparation with Standard PCR kit (KAPA Biosystems, Wilmington, MA, USA) protocol with a modified 8-bp index. Approximately 1 μg of double-stranded DNA was sheared by using a Sonicman sonicator (Brooks Automation, Spokane, WA, USA) to an average insert size of 650 bp, and DNA libraries were prepared for Illumina paired-end sequencing as described by the manufacturer. All 36 libraries were sequenced to a read length of 100 bp on the Illumina HiSeq 2500 system. Whole-genome sequence read files were deposited in the National Center for Biotechnology Information Sequence Read Archive under BioProject PRJNA291140.

### Genome Assembly

We assembled raw sequencing reads from isolate CDC-B10657 using ABySS as a reference genome for *S. kiliense* (*9*). The assembly and its corresponding read data were subject to self-alignment to determine coverage. Contigs were filtered for length and coverage; contigs <200 bp were removed from samples assembled from HiSeq data. Contigs with coverage <20% of the average coverage of the 20 largest contigs per assembly were manually removed. We assessed the final assembly for erroneous sites using Pilon (*10*).

### SNP Variant Detection and Phylogenetic Analysis

We applied a reference-based analysis method to determine genetic relatedness among all isolates. Read data of all samples were aligned to a reference by using Novoalign 3.00.03 (Novocraft Technologies, Selangor, Malaysia), and SNPs were identified by using the Genome Analysis Toolkit version 2.4 (*11*) from a custom pipeline, NASP (Northern Arizona SNP Pipeline, http://tgennorth.github.io/NASP/). The SNP calls were filtered in the final step of the pipeline and were included in the final matrix if they were not identified in repetitive regions, found in <90% of the base calls, and had a minimum read depth coverage of 10×. We excluded reads that mapped to multiple locations within the genome, as well as insertions and deletions. Only SNP loci present in all 36 isolates were included. This final matrix was created from NASP output and converted to FASTA format by using an in-house script NASP. We conducted genomewide SNP-based phylogenetics analyses using the simple parsimony algorithm in PAUP version 4.0b10 (*12*).

## Results

The total length of the de novo assembled reference contigs we showed the genome size of *S. kiliense* was ≈38 MB. The average sequencing depth for the reference strain was 354×, which resulted in 98% coverage across the genome. The assembly contained 1,092 contigs ranging in length from 500 bp to 1.7 million bp (average contig length 35,528 bp; N50 591,374 bp) (*13*).

Reference-based phylogenetic analysis identified ≈117,000 shared SNPs, of which ≈60% were parsimoniously informative (Figure). The cladogram showed 1 distinct clade that comprised all outbreak isolates and included isolates from the patients and the contaminated drugs. No more than 5 SNPs were detected between any patient and drug isolates from Chile and Colombia, demonstrating that these isolates had nearly indistinguishable genomes. Conversely, all control isolates from different sources clustered separately from the outbreak clade.

**Figure:**
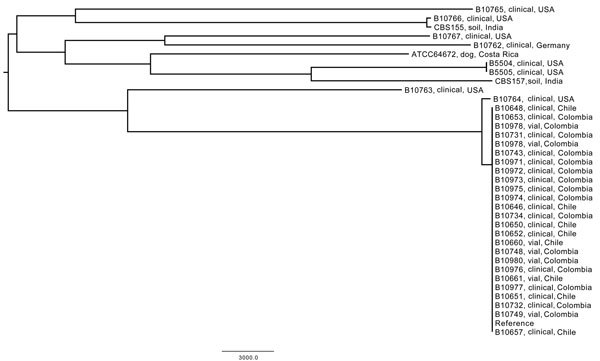
Whole-genome single-nucleotide polymorphism (SNP) typing of *Sarocladium kiliense* strains, Chile and Colombia, 2013–2014. All patient (clinical) and drug (vial) isolates from these 2 countries differed by <5 SNPs, and >21,000 SNPs were identified for the control isolates (≈117,000 total SNPs, ≈73,000 parsimoniously informative SNPs). Scale bar indicates nucleotide substitutions per site.

The genomewide SNP analysis showed greater diversity among the control isolates. Although the closest control isolate, B10764, differed from the outbreak clade by 501 SNPs, the remaining majority of the control isolates diverged from the outbreak clade by ≈21,000 SNPs. Two control isolates, B5504 and B5505, isolated from a 1996 cluster of *S. kiliense* infections in a US hospital, were genetically indistinguishable from each other.

## Discussion

*S. kiliense* is primarily a saprobic soil organism; it can cause opportunistic infections in humans that typically occur after inoculation of the fungus (*14*). Here we report the results of molecular epidemiologic investigation of an outbreak of *S. kiliense* BSI that affected >50 patients in 2 South American countries. The epidemiologic investigation suggested contaminated antinausea medication as a possible source of this infection. With no existing genotyping methods for this uncommon pathogen, we used WGST to understand the genetic relationships among the isolates and identify a potential source of this outbreak.

The use of WGST to investigate fungal outbreaks has become integral to epidemiologic investigations (*3*–*5*,*15*). During nosocomial outbreak investigations, ascertaining potential source(s) of infection based solely on the descriptive epidemiologic findings often is difficult. Molecular genotyping frequently is needed to test hypotheses generated by epidemiologic investigation: the presence of a single strain or dominant clone usually suggests a point source, whereas the presence of multiple strains is usually consistent with an environmental exposure or exposure to mixed populations from a single source. For example, in a recent outbreak of *Curvularia* spp. (formerly *Bipolaris* spp.) among cardiac surgery patients, initial epidemiologic investigation suggested a point source; however, a molecular epidemiology analysis demonstrated multiple strains, consistent with an environmental source (*16*,*17*). Similarly, in an outbreak of *Fusarium* spp. associated with the use of contact lenses, initial epidemiologic investigation linked the infections with a particular lens cleaning solution, suggesting a point source; however, molecular analysis indicated multiple sources (*18*). In our current investigation, the WGST analysis demonstrated that the patient isolates from Chile and Colombia were nearly genetically indistinguishable (<5 SNPs) from those recovered from the medication vials, indicating the likely presence of a single-source infection. Conversely, the control isolates clustered differently from the outbreak clade by ≈21,000 SNPs, and except for 2 strains from the same cluster, thousands of SNPs separated any 2 control strains (Figure).

Although BSIs with this organism are rare, case reports/clusters of *S. kiliense* fungal infections have been reported in the literature (*19*–*22*). In a cluster of *S. kiliense* infections of endophthalmitis and catheter-related BSIs, an environmental source was strongly suggested but could not be confirmed because of the lack of available typing methods (*21*). Isolates from this cluster of infections served as controls in our study: specifically, isolates B5504 and B5505 from 2 patients who underwent cataract extraction with intraocular lens implantation differed from each other by 1 SNP, indicating that the 2 genomes were nearly indistinguishable and suggesting a common source of infection.

In this investigation, the use of WGST identified a likely source of *S. kiliense* BSI in oncology patients in both countries by linking these infections to the receipt of contaminated medication. Results from this outbreak are consistent with those from other outbreaks of fungal pathogens in which WGST was used and a common source was hypothesized. For example, Litvintseva et al. investigated *Exerohilium rostratum* infections associated with the injection of contaminated methylprednisolone acetate by using whole-genome SNP typing; they found that all outbreak isolates were genotypically indistinguishable: no more than 2 SNPs separated the strains in the outbreak clade (*3*).

The genetic diversity among the *S. kiliense* control strains in our study was congruent to the level of diversity among control isolates found in other studies. We identified comparable levels of genetic similarities to those found among isolates of *Saprochaete clavata* from patients from a multicenter outbreak in France (*15*). Conversely, >28,000 and up to 1.2 million SNPs were observed among the control isolates of *Apophysomyces trapeziformis* infections associated with a tornado in Joplin, Missouri, USA (*5*). Although we were able to include a variety of controls from different sources, a major limitation in this study is the lack of background isolates of *S. kiliense* from the affected countries to assess genomic similarity among the unrelated isolates from South America.

Traditional molecular strain typing methods, such as MLST, are limited to analysis of specific genomic regions, typically protein-coding regions. However, the conservation in these regions might be insufficient to discriminate strains of certain fungi or provide the resolution needed to identify a likely source in a fungal outbreak. In the absence of established methods for fungi, the rapid development of traditional typing methods is often necessary. In this investigation, before deploying WGST, we evaluated MLST using 7 protein-coding regions to determine the relatedness among all isolates (*17*). Across 3,651 nt amplified, only a 1-nt polymorphism was identified in the β-tubulin gene of one of the control isolates, which did not provide sufficient resolution between outbreak and control isolates, indicating that the use of MLST was not informative in this outbreak investigation. Although conventional typing methods are rapid, the conclusions drawn from them might be confounded by either their discriminatory power or their character state conflict (i.e., homoplasy). Typically, genotyping methods with high resolution (e.g., variable-number tandem-repeat typing) have higher degrees of homoplasy, whereas methods with lower resolution (e.g., MLST) have less homoplasy; both might be at risk of misidentifying a common source. Conversely, WGST enables accurate deduction of genetic relationships among strains in fungal outbreaks for which current typing methods are ineffective or nonexistent. Furthermore, with the improvement of next-generation sequencing technologies, investigating fungal outbreaks in real time will soon be possible.

Contamination of medical products, particularly with rare fungi, poses growing concern and a public health threat, especially in vulnerable populations. Fungal clusters/outbreaks of common environmental species (rather than classical clinical pathogens) have been associated with medical products. For example, the 2002 outbreak of fungal meningitis was caused by injected steroids contaminated with *Exophiala dermatitidis*, and the 2012–2015 outbreak of fungal meningitis and other infections resulted from methylprednisolone contaminated by *Exserohilum rostratum* (*3*,*23*). Similarly, in 2009, intestinal zygomycosis resulted from ingestion of allopurinol tablets contaminated with *Rhizopus microsporus* (*24*). In 2012, an endophthalmitis outbreak was associated with use of an ophthalmologic dye contaminated with *Fusarium* sp. in the *Fusarium incarnatum-equiseti* species complex and triamcinolone contaminated with *Curvularia hawaiiensis* (*Bipolaris hawaiiensis*) (*16*,*25*,*26*). Increased vigilance and the use of advanced technologies are needed to rapidly identify the likely source(s) of infection to efficiently guide epidemiologic investigations and initiate appropriate control measures. 

In summary, our study highlights the utility of advanced molecular methods to investigate outbreaks involving rare fungi. Next-generation sequencing and bioinformatics analyses will remain critical molecular epidemiology tools in such epidemiologic investigations.
